# Supplementary material for: Empirical methods for controlling false positives and estimating confidence in ChIP-Seq peaks
Source: BMC Bioinformatics. 2008 Dec 5;9:523. doi: 10.1186/1471-2105-9-523 (PMC2628906; doi:10.1186/1471-2105-9-523)
Supplement: Additional File 1 — A variety of html documents from the USeq web site detailing the available applications, their best usage, output file type descriptions, command line menus, etc. [file 1471-2105-9-523-S1.zip › USeqUserGuides/JavaDoc.zip!/JavaDoc/trans/cel/package-summary.html]

trans.cel


|  |  |  |
| --- | --- | --- |
| **Overview** \| **Package** \| Class \| **Use** \| **Tree** \| **Deprecated** \| **Index** \| **Help** | |  |
| **PREV PACKAGE**   **NEXT PACKAGE** | **FRAMES**    **NO FRAMES**     **All Classes** |  |


---

## Package trans.cel

Apps and methods for manipulating cel files.

**See:**
  
          **Description**

| **Class Summary** | |
| --- | --- |
| **AverageACels** | Averages two serialized float[][]s, like xxx.cela files, from the CelFileConverter app. |
| **AveragePCels** | Averages serialized float[]s, like xxx.celp files, from the CelProcessor app. |
| **CelFileConverter** | Saves text cel files as serialized float[][]s for downstream use by TiMAT applications. |
| **CelLine** | Class for holding info about a text cel file line, enables sorting after rotation. |
| **CelMapper** | Saves float[]s of intensity values for each cel file ordered by the tpmapFeature file. |
| **CelPHistogram** |  |
| **CelProcessor** | Application to process '.cela' files, wrapper for `CelMapper`, `QuantileNormalization`. |
| **ChromosomeIndex** | Use to keep track of intensities in a array, their chromosome name, and start stop indexes. |
| **ComparatorMultiSetQuantile** | For sorting MultiSetQuantile objects by value or chromosome number and position |
| **ComparatorQuantile** | For sorting ComparatorQuantile objects by value or chromosome number and position |
| **ControlStats** |  |
| **ConvertTVSToCelps** | Converts a tsv file to xxx.celp files (float[]s) linked to the tpmap. |
| **MakeChromosomeSets** |  |
| **MultiSetQuantile** | Class holding info relating to one quantile value. |
| **Quantile** | Class holding info relating to one quantile value. |
| **QuantileNormalization** | Performs quantile normalization on arrays of double values. |
| **RotateCelFile** |  |
| **SplineScalar** | Generates multiplicative scalars based on a set of points using a combination of a cubic spline for values within the provided points and linear regression for points outside the value range. |
| **StatIntensityFeatureArray** |  |

## Package trans.cel Description

Apps and methods for manipulating cel files.

---


|  |  |  |
| --- | --- | --- |
| **Overview** \| **Package** \| Class \| **Use** \| **Tree** \| **Deprecated** \| **Index** \| **Help** | |  |
| **PREV PACKAGE**   **NEXT PACKAGE** | **FRAMES**    **NO FRAMES**     **All Classes** |  |


---
